# *Tityus serrulatus* Scorpion Venom Induces Apoptosis in Cervical Cancer Cell Lines

**DOI:** 10.1155/2019/5131042

**Published:** 2019-06-23

**Authors:** Emanuelly Bernardes-Oliveira, Kleber Juvenal Silva Farias, Dayanne Lopes Gomes, Josélio Maria Galvão de Araújo, Wilmar Dias da Silva, Hugo Alexandre Oliveira Rocha, Eduardo Antônio Donadi, Matheus de Freitas Fernandes-Pedrosa, Janaina Cristiana de Oliveira Crispim

**Affiliations:** ^1^Programa de Pós-Graduação em Desenvolvimento e Inovação Tecnológica em Medicamentos, Universidade Federal do Rio Grande do Norte, Natal, RN, Brazil; ^2^Programa de Pós-Graduação em Ciências Farmacêuticas, Universidade Federal do Rio Grande do Norte, Natal, RN, Brazil; ^3^Departamento de Bioquímica, Centro Biociências, Universidade Federal do Rio Grande do Norte, Natal, RN, Brazil; ^4^Departamento de Microbiologia e Parasitologia da Universidade Federal do Rio Grande do Norte, Natal, RN, Brazil; ^5^Laboratorio de Virologia, Instituto de Medicina Tropical, Universidade Federal do Rio Grande do Norte, RN, Brazil; ^6^Instituto Butantan, Laboratório de Imunoquímica, São Paulo, SP, Brazil; ^7^Faculdade de Medicina de Ribeirão Preto, Universidade de São Paulo, Ribeirão Preto, São Paulo, Brazil; ^8^Maternidade Escola Januário Cicco (MEJC), Natal, RN, Brazil

## Abstract

Cervical cancer (CC) is classified as the fourth most common type of cancer in women worldwide and remains a serious public health problem in many underdeveloped countries. Human papillomavirus (HPV), mainly types 16 and 18, has been established as a precursory etiologic agent for this type of cancer. Several therapeutic attempts have been studied and applied, aiming at its control. However, not only do classical treatments such as chemotherapies and radiotherapies target tumor cells, but also they cause damage to several healthy cells. For these reasons, the search for new biologically active chemotherapeutic components is of great importance. In this study, we investigated the effect of* Tityus serrulatus* scorpion venom (TsV) on CC lines. There are very few studies exploring venom of scorpions, and, to our knowledge, no study has been conducted using the venom of the scorpion TsV for treatment of cervical cancer lines. After challenge with TsV, the MTT assay demonstrated cytotoxic effect on HeLa line. Similarly, the cell death process in HeLa analyzed by flow cytometry suggests death via caspase, since the pan-caspase inhibitor z-VAD-fmk significantly reduced the apoptotic response to the treatment. These results suggest that venom of TsV can be a potential source for the isolation of effective antiproliferative and apoptotic molecules in the treatment of CC.

## 1. Introduction

Cervical cancer (CC) is the fourth most common female malignancy worldwide [1], responsible for 7.5% of all female cancer deaths [2]. In Brazil CC remains a major health problem and is considered the third most frequent type of cancer among Brazilian women, excluding nonmelanoma skin tumors [3]. According to the Brazilian National Cancer Institute, there is an estimated incidence of 16.370 cases per 100,000 women for the biennium 2018-2019 [4]. Persistent infection of oncogenic human papillomavirus (HPV) is strongly associated with risk of cervical cancer and genital warts [5], with about 40 types being sexually transmitted and approximately 15 types being classified as being high risk for cervical oncogenesis [6, 7]. Types 16 and 18 are the most prevalent and are responsible for 70% of cervical cancer cases [8].

Currently, treatments for this type of cancer are surgical removal via tumor radical hysterectomy [9], cisplatin-based chemotherapy sessions [2], uterine cavity brachytherapy, or intensity-modulated radiotherapy [10]. These treatments with chemotherapy and radiation can create tumor cells that become resistant [11] and the death of healthy cells which consequently leads to side effects, preventing their effectiveness [12].

Moreover, chemotherapy has been shown in some cases to have a limited effect on the cure and survival of patients, particularly in patients with cervical cancer, and especially when the disease is advanced; what is more it may promote chemoresistance or even relapse, which limits the success [13–15]. Therefore, an urgent goal in medicine is the search for new biomarkers with lower cytotoxicity, with less side effects, and, at the same time, selective with normal cells, or at least presenting minimal effects [16, 17].

With this, one of the biggest challenges today for medicine is the search for a more effective treatment for cancer, including CC. Recently, studies have shown that venom of arthropods is a promising source in the fight against cancer [18, 19]. Therefore, the search for new natural-born therapies has been extensively studied, from which scorpion venoms have shown much promise [20, 21]. Previous studies, with the species* Androctonus crassicauda *and* Leiurus quinquestriatus *with their use as an antitumor agent, showed that they contributed to cell death of MDA-MB-231 (human breast cancer) and HCT-8 (colorectal cancer) and induced reduced cell motility and colony formation correlated with the inhibitory role of scorpion venom [22]. While Diaz-García [23] demonstrated that the crude venom of* Rhopalurus junceus* scorpion promoted high cytotoxicity and apoptosis via the mitochondrial line MDA-MB-231. In another study, using fractions from the venom of another scorpion species,* Androctonus australis hector*, it was observed that the F3 fraction of the venom presented cytotoxic action in human lung cancer cells (NCI-H358), inducing death by oxidative stress and mitochondrial dysfunction [12]. In a previous study from our group of researchers, we observed that an isolate scorpion venom of the species* Tityus stigmurus*, showed cytotoxicity in SiHa cells [24]. There are few studies exploring the venom of scorpions, and, to our knowledge, no study has been conducted using the venom of the scorpion* T. serrulatus* in cervical cancer lines. Herein, we investigated the antiproliferative effects of TsV scorpion venom in cervical cancer cells.

## 2. Material and Methods

### 2.1. Scorpion Venoms

The crude venom of* Tityus serrulatus* (TsV) was provided by Arthropods Laboratory, Institute Butantan, Brazil. The venom was obtained by electrostimulation from the method of Bucherl (1969) with slight modifications. Briefly, 15 – 20 V electrical stimuli were repeatedly applied to the scorpion telson and the venom drops were collected with a micropipette, and lyophilized. Stock solutions of crude venoms (500 *μ*g/mL) were prepared in DMEM culture medium and filtered using a 0.22 *μ*m of millipore membrane (TPP Techno Plastic Products AG, Trasadingen, Switzerland). The use of TsV scorpion venom was developed under authorization of “Brazilian Access Authorization and Dispatch Component of Genetic Patrimony (CGEN)” (Process 010844/2013-9, 25 October 2013).

### 2.2. Cell Lines and Reagents

SiHa human squamous cell carcinoma HPV-16 and HeLa-18 cervical adenocarcinoma cells HPV-18-positive were donated by Dr Ana Paula Lepique (Department of Immunology, University of São Paulo, Brazil).

The cells were cultured in Dulbecco's modified Eagle's medium (DMEM, Cultilab, Campinas, SP, Brazil), supplemented with 10% fetal bovine serum (FBS, Cultilab, Campinas, SP), sodium pyruvate, and essential amino acids (Sigma-Aldrich, St. Louis, MO, USA), and 1% penicillin/streptomycin solution (Life Technologies, California, EUA) at 37 C, 5% CO_2_.

MTT 3-(4.5-dimethylthiazol-2-yl)-2-5-diphenyltetrazolium bromide and inhibitor z-VAD-fmk (carbobenzoxy-valyl-alanyl-aspartyl-[O-methyl]-fluoromethylketone) were obtained from Sigma-Aldrich (St. Louis, MO, USA), Annexin V-FITC/Propidium iodide (PI) kit Invivogen (San Diego, USA) and DMSO P.A (Dimethyl sulfoxide) came from Sigma-Aldrich, (St. Louis, MO, USA). CDDP (Cisplatin, citoplax), 50 mg, was obtained from Bergamo Taboão da Serra, SP, Brazil.

### 2.3. Cell Culture and MTT Colorimetric Assay

SiHa and HeLa cells were cultivated in a 96-well plate at an initial density of 5 × 10^3^ cells/well and after 24 h were treated with 200 *μ*L/well of different concentrations of TsV venom (50, 125, 250, and 500 *μ*g/mL) and CDDP 33 *μ*g/mL [16] [Cisplatin, citoplax, 50 mg from Bergamo Taboão da Serra, SP, Brazil, drug control]. During the procedures we used as negative control (NC) cells with only culture medium. All concentrations were used in triplicate and incubated for 24 h and 48 h, with the function of determining venom cytotoxicity by MTT colorimetric assay at 5 mg/mL (50 *μ*L/well) in nonsupplemented culture medium and incubated for 4 h at 37°C. Then, the medium was removed and the precipitated formazan crystals were dissolved in 100 *μ*L of DMSO. After 15 min, the MTT reduction was analysed by measuring the absorbance at 540 nm in a microplate reader (Biochrom® Asys Expert Plus), which was used for data analysis as described by Bernardes-Oliveira et al., 2016. The absolute value of MTT reduction was calculated as follows:

(1) $$\begin{array}{c} MTT\;\;reduction=\frac{Abs.\;\;540\;\;nm\;\;of\;\;sample}{Abs.\;\;540\;\;nm\;\;of\;\;control} \end{array}$$

### 2.4. Annexin V-FITC/PI Double Staining and Analysis by Flow Cytometry and zVAD-fmk

Finally, to evaluate the effects of TsV venom on cell death of tumor cells, the SiHa and HeLa cells were cultivated in 6-well plates (2 × 10^5^ cells/well), after treatment with 1 mL TsV venom 250 *μ*g/mL where there were no differences between the concentrations of 250 *μ*g/mL and 500 *μ*g/mL of the venom when the HeLa line was treated (see Figure 2(b)). After 48 h, the medium was removed and the cells were incubated with 5 *μ*L Annexin V-FITC and 1 *μ*L Propidium Iodide (PI); then the mixture was incubated for 15 min in the dark at room temperature following kit directions (Invitrogen, Catalog numberV13242) as described in [25]. In the caspase activity assay, the 10 *μ*M zVAD-fmk caspase inhibitor was used in the presence of TsV, for the other procedures the same methodology for the Annexin and PI assay of the previous experiment was used. The cells were analyzed by flow cytometry (flow cytometer FASCANTO II, BD Biosciences), measuring fluorescence emission at 530–575 nm for annexin V and 630/22nm for PI. 10,000 events were acquired. The FlowJo software version X10.0.7 (Tree Star, Inc., Ashland, OR, USA) was used for data analysis.

### 2.5. Statistical Analysis

Each experiment was performed at least 3 times. We used analysis of variance (ANOVA) and Tukey's *t*-test. Differences with *p* < 0.001 between the values are considered statistically significant. Statistical analysis and the Pearson correlation coefficient (*ρ*) were performed using GraphPadInStat_software version 4.0 (GraphPad software, San Diego, CA, USA).

## 3. Results

### 3.1. Cell Morphology after Treatment with Scorpion Venom

**Figure 1:**
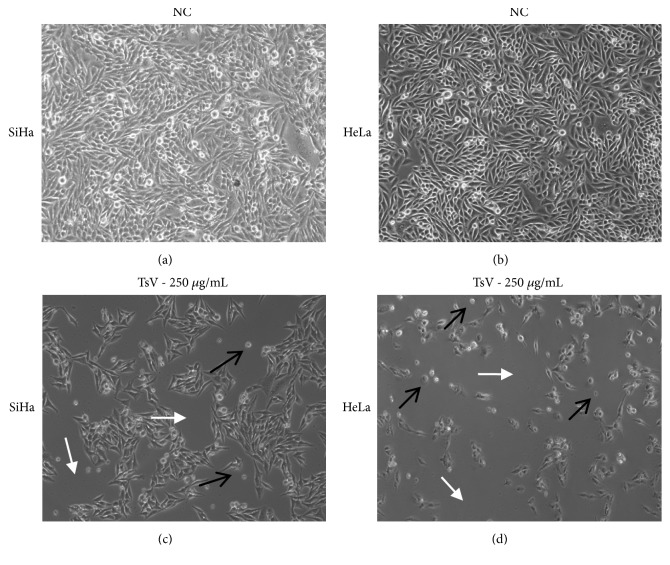
*Morphological alterations of SiHa and HeLa cells induced by Tityus serrulatus venom.* SiHa and HeLa cells were treated with extract (250 *μ*g/mL) for 48 h. After incubation, the cells were examined under light microscopy. The data are a representative example for duplicate tests. Magnification ×10.

SiHa e HeLa cells were exposed to TsV (250 *μ*g/mL) for 48 h. Subsequently, their morphology was analyzed. In Figures 1(a) and 1(b) images of the SiHa and HeLa cells (negative control) and in Figures 1(c) and 1(d) images of cells treated with the venom can be observed. The negative control cells exhibited a typical SiHa (HPV-16) or HeLa (HPV-18) cell morphology in culture, i.e., confluent monolayer with homogeneous, slightly triangular cells, normal nuclear and cytoplasmic appearance, and a few floating cells that have not adhered to the culture vessel. On the other hand, following the addition of TsV, the number of tumor cells was reduced, and the characteristic confluent monolayer was abrogated (Figures 1(c) and 1(d), white arrow), presenting cytoplasmic retraction (Figures 1(c) and 1(d), black arrow).

### 3.2. Cytotoxic Effect of Tityus serrulatus Venom

**Figure 2:**
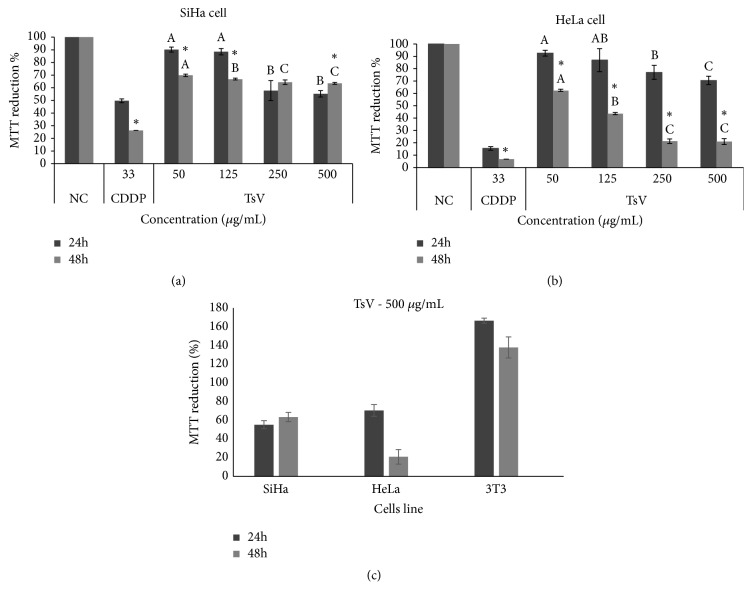
*Cytotoxicity of Tityus serrulatus scorpion venom in cervical cancer lineage SiHa (a) and HeLa (b), 24h and 48h.* Negative control group (NC) and* T. serrulatus* (TsV). Cisplatin (CDDP) was used as control drug. Different letters  ^a,b,c^ indicate significant differences between treatments with the same kinetics (*p <0.001*),  ^*∗*^ indicates the same concentrations for different kinetics (*p <0.001*). 3T3 line (normal cell), treated at the highest concentration, compared to the CC lineages (c). The results were analyzed using ANOVA-Tukey test.

**Figure 3:**
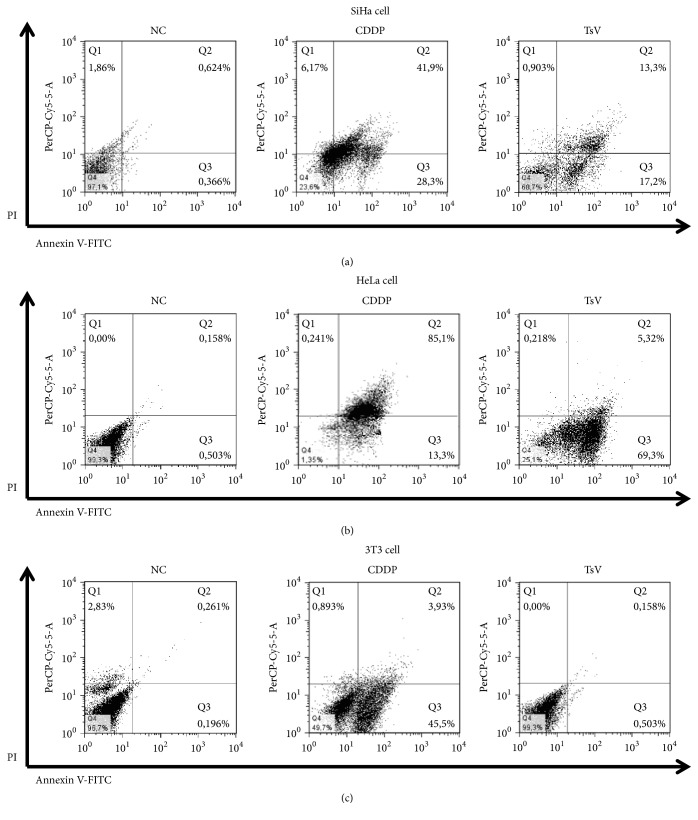
*Flow cytometry analysis of HeLa cells after exposure to T. serrulatus scorpion venom.* SiHa (a), HeLa (b), and 3T3 (c) lines, treated with 250*μ*g/mL* T. serrulatus* (TsV). Annexin−/PI−(Q4), viable cells; Annexin+/PI−(Q3), cells undergoing apoptosis; Annexin+/PI+(Q2), cells that are in end-stage apoptosis or are already dead; Annexin−/PI+(Q1), cells that are in necrosis.

The crude venom of the scorpion was used in different concentrations (see Methods) in order to analyze the TsV venom cytotoxic action on the growth of the SiHa and HeLa cervix cancer lines (Figures 2(a) and 2(b)) and 3T3. When we treated the tumoral cell lines with TsV 250 *μ*g/mL for the duration of 48 h, the SiHa line presented cytotoxicity of 64.36% when compared to CDDP 26.23% and the NC group did not show cytotoxicity (Figure 2(a)). In relation to cytotoxicity of TsV in the HeLa line, a dose-dependent cytotoxic response of 78.7% was observed compared to CDDP (93.2%), and the NC group did not show cytotoxicity (Figure 2(b)). The same treatment in the normal 3T3 line was not cytotoxic (Figure 2(c)).

### 3.3. Cytometry Analysis of SiHa and HeLa Cells after Exposure to TsV Venom

To determine the cell death pathway induced by TsV venom, the SiHa, HeLa, and 3T3 cells were tested for detection by Annexin and PI double staining. Generally, cells labeled with Annexin indicate initial apoptosis, cells labeled with PI are indicative of necrosis, and cells positive for Annexin and PI are indicative of late apoptosis [16, 25]. The mechanism of cell death by apoptosis is probably the most effective action against progression of tumors and most therapeutic drugs, such as cisplatin (CDDP), which is considered the gold standard treatment, inhibiting proliferation of cancer cells via apoptosis. In order to exploit the cytotoxic effect of scorpion venom by flow cytometry, TsV venom 250 *μ*g/mL concentrations were applied in SiHa and HeLa human cervical carcinoma lines and in normal 3T3 cells (Figure 3). When we evaluated apoptosis during treatment with TsV, it was observed that the SiHa line presented 30.5% of apoptosis when compared to CDDP 70.2% and the NC group 0.99% (Figure 3(a)). In regard to the HeLa line, TsV induced 74.6% death by apoptosis when compared to CDDP 98.4% and NC group 0.66% (Figure 3(b)). To confirm the selectivity of TsV venom for only tumor cells, we used the normal 3T3 lineage, which did not present posttreatment apoptosis and showed 99.3% viability, when compared to 49.7% CDPP and 96.7% NC group.

### 3.4. Caspase Inhibitor Activity Assay

**Figure 4:**
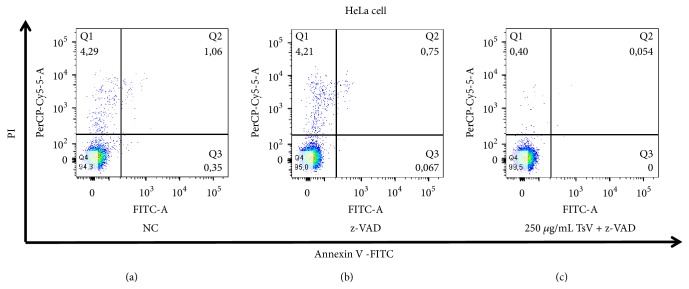
*Apoptosis assay HeLa cells after exposure to TsV and z-VAD-fmk.* HeLa cells* treated with 250μg/mL* TsV with the caspase inhibitor. Annexin−/PI−(Q4), viable cells; Annexin+/PI-(Q3), cells undergoing apoptosis; Annexin+/PI+(Q2), cells that are in end-stage apoptosis or are already dead; Annexin−/PI+(Q1), cells that are in necrosis.

In view of the results analyzed in the previous experiment with the HeLa cells and to confirm whether death was via caspase, we used z-VAD-fmk in the presence and absence of TsV and found that there was inhibition of z-VAD-fmk when treated with 250 *μ*g/mL of venom (Figure 4(c)).

## 4. Discussion

The high incidence of cervical cancer affects women all over the world. What is more, mortality from this type of cancer has gradually increased in various countries. The discovery of molecular signatures in cancer, with the potential for future biomarker development to identify individuals who are at high risk of cancer, is still needed in clinical practice. Further studies are warranted to evaluate different approaches and explore the predictive potential, especially in the detection of individuals who are vulnerable to progression of intraepithelial lesions and cervical cancer. In general, many of the commonly used serum tumor biomarkers are limited to late-stage disease and have low sensitivity and specificity. It is known that chemotherapy is a common therapeutic intervention for different types of cancer [26], including cervical cancer, and is used as primary or adjuvant therapy.

However, these types of chemotherapeutic interventions can cause several side effects to the patient, promote resistance to the tumor, and are not selective, causing damage to normal cells [27].

That is why using scorpion venom as a source represents an interesting biological treatment for cancer ([28]; Giovannini et al., 2017). Herein, we investigated the antiproliferative effects of TsV, and the treatment with these venoms resulted in a dose-dependent decrease in HeLa cell viability. The same was not observed in the SiHa cells, demonstrating a lower sensitivity. Studies by Machado et al. (2017) found that the multifunctional antimicrobial peptide of the* T. stigmurus* scorpion (TistH) has a cytotoxic action for SiHa cells; the same was not found when normal 3T3 cells were challenged under the same conditions.

In view of these findings, the TsV venom in HeLa cells is classified as a pioneer, even in other tumor strains. Recently Luo et al. [29] demonstrated that about 60-70% of this tumor lineage was resistant to chemotherapy with cisplatin when compared to the sensitivity of HeLa and C33A cells. Evidence emerging from this study has shown that 78 kDa glucose-regulated protein (GRP78) may be the inducer of chemoresistance in SiHa and to strengthen this break in resistance, it suggests the silencing of GRP78 which plays a significant role in the progress of oncogenesis. This approach can hypothesize that this susceptibility differential may be related to the expression of the HeLa cell, recognized by the TsV venom. Indeed, as seen by Contreras-Ortiz and colleagues [30], venom of the scorpion* Centruroides limpidus limpidus* was not cytotoxic to HeLa cell cultures, suggesting that this may be partially attributable to the absence of specific cell membrane targets for molecules present in the venom.

One of the mechanisms of death induced by scorpion venoms is via caspases, the beneficial pathways in cancer therapy, as seen in our results, corroborating with the studies of other researchers who challenged different tumor cell lines, including leukemic cells when treated with the venom of the scorpion* Heterometrus bengalensis Koch* [31], cervical cancer cells (*Rhopalurus junceus*) [28], and prostate cancer cells (*Androctonus amoreuxi*) (Akef et al. 2017). These results were also seen by Al-Asmari et al. [32], where venoms of the scorpions* Androctonus crassicauda, Androctonus bicolor, *and* Leiurus quinquestriatus* also induced a death via apoptosis. Therefore, based on these findings and other published studies, a conclusion can be drawn that scorpion venom possesses cytotoxic and apoptotic properties against cervical cancer cell lines. This type of death was confirmed when the pan-caspase inhibitor (zVAD-fmk) was activated, in the presence of TsV venom, suggesting that the venom of this species of scorpion may be a strong candidate for antitumor treatment. To our knowledge, no approaches demonstrate that* T. serrulatus* scorpion venom possesses cytotoxic properties against cervical cancer cell lines.

## 5. Conclusions

Studies with scorpion venom have contributed significantly to the development of new biomedical research. Various bioactive molecules can be found in these venoms, which may present significant pharmacological activity in human physiology. More research is needed to better understand the cytotoxic action of* Tityus serrulatus* venom. Therefore, we suggest studying more deeply the mechanisms involved in cell death. So far the results are promising for its application in cervical cancer therapy.

## Data Availability

The data used to support the findings of this study are available from the corresponding author upon request.
